# Combined (d)SPE-QuEChERS Extraction of Mycotoxins in Mixed Feed Rations and Analysis by High Performance Liquid Chromatography-High-Resolution Mass Spectrometry

**DOI:** 10.3390/toxins12030206

**Published:** 2020-03-23

**Authors:** Rocio Facorro, Maria Llompart, Thierry Dagnac

**Affiliations:** 1 Galician Agency for Food Quality—Agronomic and Agrarian Research Centre (AGACAL-CIAM), Unit of Organic Contaminants, Apartado 10, 15080 A Coruña, Spain; rocio.facorro@rai.usc.es; 2 Laboratory of Research and Development of Analytical Solutions (LIDSA), Department of Analytical Chemistry, Nutrition and Food Science, Faculty of Chemistry, E-15782 Campus Vida, Universidad de Santiago de Compostela, 15782 Santiago de Compostela, Spain; maria.llompart@usc.es

**Keywords:** Mycotoxins, mixed feed rations, QuEChERS, dispersive solid phase extraction, liquid chromatography, high-resolution mass spectrometry, data independent SWATH

## Abstract

The objective of this work was the development of a methodology capable of simultaneously determine 26 mycotoxins in mixed feed rations collected in 20 dairy farms. A sample preparation methodology based on a combination of (d)SPE and QuEChERS extractions was used. Liquid chromatography-high resolution mass spectrometry was employed for both identification and quantification purposes. To this respect, a powerful workflow based on data-independent acquisition, consisting of fragmenting all precursor ions entering the mass spectrometer in narrow m/z isolation windows (SWATH), was implemented. SWATH data file then contains all the information that would be acquired in a multitude of different experimental approaches in a single all-encompassing dataset. Analytical method performance was evaluated in terms of linearity, repeatability and matrix effect. Relative recoveries were also measured, giving values above 80% for most compounds. Matrix-matched calibration was carried out and enabled reaching the low ng mL^−1^ level for many mycotoxins. The observed matrix effect, in most cases suppressive, reached even values higher than 60%. The repeatability was also adequate, showing a relative standard deviation lower than 10%. All unified samples analyzed showed co-occurrence of two or more mycotoxins, recurrently zearalenone, fumonisin B1, and β-zearalenol, with an occurrence frequency ranging from 60% to 90%.

## 1. Introduction

Mycotoxins are natural secondary metabolites produced by various filamentous fungi, mainly belonging to *Aspergillus*, *Penicillium,* and *Fusarium* genera. Around 400 of these metabolites are identified and classified, being the most predominant zearalenone, ochratoxin A, fumonisins and trichothecenes [1]. Mycotoxins can be produced during all stages; from pre-harvest, when plants are growing, to storage, transport or further processing [2].

Mycotoxins can cause some adverse effect on animal and human health. They can be carcinogenic agents (aflatoxin B_1_ (AFB_1_), fumonisins B1 and B2, (FB_1_ and FB_2_)), present estrogenic activity (zearalenone (ZEA)), or teratogenic (ochratoxin A, (OTA)) and immunotoxinogenic effects (deoxynivalenol, (DON)) [3]. In consequence, the presence and intake of mycotoxins can also strongly reduce animal productivity in farms. The severity of the damage depends on the compound, the degree of exposure and the possible co-occurrence of various mycotoxins. This is a frequent situation, such is the case with zearalenone and deoxynivalenol or zearalenone/deoxynivalenol/nivalenol/fumonisin B_1_ [4].

Most studies concerning mycotoxins occurrence were carried out in food matrices, cereals [5,6,7,8] feedstuff [1,9,10], plant based beverages [11], and ruminant milk [12]. Nonetheless, some researchers also found traces of these toxins in human breast milk [13,14]. Due to mycotoxins stability to high temperatures and persistence during food preparation procedures [15], they present a risk to the whole food chain.

With the purpose of protecting consumers against mycotoxin intake, maximum levels of aflatoxins (AFB1, and sum of AFB1, AFB2, AFG1, and AFG2), sum of FB1 and FB2, DON, OTA, and ZEA, were established by the European Union for products intended for animal feeding [16,17]. In 2013, maximum recommended levels were set for the sum of toxins T-2 and HT-2 in cereals, as well [18].

Due to the hazard mycotoxins may cause, sensitive and selective analytical techniques are needed for their monitoring in food and feed. Besides, because of their frequent co-occurrence, the European Food Safety Authority (EFSA) recommends multianalyte methodologies. Nevertheless, finding an appropriate methodology, in terms of sample preparation, for a wide number of mycotoxins can be a tough task due to the structural variety and different chemical properties of the compounds.

Many sample treatment procedures for the simultaneous determination of multiple mycotoxins have been published. From classic approaches like solid liquid extraction (SLE) and liquid–liquid extraction (LLE) to the most recent ones, like modified Quick, Easy, Cheap, Effective, Rugged, and Safe (QuEChERS) extraction or matrix solid-phase dispersion (MSPD). Modified QuEChERS procedure is the sample pre-treatment of choice in many recent publications, due to its simplicity and cost-efficiency [8,9,19,20].

In the case of food and feed samples, clean-up steps are usually needed in order to reduce the matrix effect, especially in the case of liquid chromatography analyses. Solid phase extraction (SPE) and dispersive solid phase extraction (dSPE) are the most used as described in literature [19,21,22,23].

In terms of analysis, two different approaches are currently employed to control the presence of mycotoxins in food and feed. For screening and semi-quantitative analysis, enzyme-linked immune-sorbent assay (ELISA) and thin-layer chromatography (TLC) are the most frequently used. To provide accurate quantitative information and therefore reduce the number of possible false-positive results, chromatographic techniques such as gas chromatography (GC) and high-performance liquid chromatography (HPLC) coupled to ultraviolet (UV), fluorescence (FL), and mass spectrometry (MS) detectors are used [24,25].

HPLC coupled to tandem mass spectrometry (MS/MS) is considered the most reliable technique in order to achieve high specificity and sensitivity for the simultaneous determination of a large number of mycotoxins, reaching low limits of detection (LOD) and quantification (LOQ). Several studies using this technique for the analysis of multi-mycotoxins in food and feed commodities have been published [9,10,12,26,27].

More recently, high-resolution mass spectrometry (HRMS) has been implemented either in Data Dependent Acquisition mode or in Data Independent Acquisition mode. Besides providing high specificity and resolution due to the use of the accurate mass, HRMS allows for performing non-target screening. Quadrupole time-of-flight (QTOF) instruments offer the advantages of TOF detectors in terms of resolution and the possibility of acquiring product ion spectra working in MS/MS mode using accurate mass. This feature provides additional confirmatory parameters besides retention time and ion fragment ratio, i.e., exact mass, isotope pattern and spectral comparison against spectra libraries with smart confirmation criteria. Among the applications published employing quadrupole time-of-flight and Orbitrap^®^ detectors [1,19,28,29] for the mycotoxin analysis in food and feed, the main part of them use Orbitrap technique.

Hence, the aim of this work was to develop a methodology that allowed the simultaneous analysis of multi-class mycotoxins in mixed rations. A QuEChERS extraction method was implemented, with the addition of a clean-up step based on solid phase extraction and dispersive solid phase extraction, in order to minimize the effect of the matrix. The analytical methodology was carried out using HPLC followed by triple quadrupole-time-of-flight detection based on a data independent workflow.

The method was optimized and validated for 26 mycotoxins produced by different genera. Several analytical parameters, such as linearity, repeatability, accuracy, matrix effect, measurement uncertainties, limits of detection, and recovery, were evaluated.

A monitoring of these mycotoxins was carried out using the proposed methodology analyzing 97 mixed ration samples collected in 20 dairy farms from Galicia (NW Spain) over one year. These rations may constitute the basis of the diet for dairy cows during 6 to 8 months of the year.

## 2. Results and Discussion

### 2.1. Chromatographic Analysis

The aim of this work was to develop an analytical methodology based on high performance liquid chromatography coupled to triple quadrupole-time of flight mass spectrometry for the simultaneous analysis of the 26 mycotoxins. Hence, the chromatographic conditions were optimized to achieve an efficient separation of the target compounds (see conditions in the Experimental section). The initial step was to consider the ionization of the target mycotoxins by injection of a standard containing all analytes, both in positive and negative mode. This allows for the selection of the most sensitive mode for each compound.

Two runs were needed for each analysis, since the methodology does not allow switching from one ionization mode to the other in such short time.

**Figure 1:**
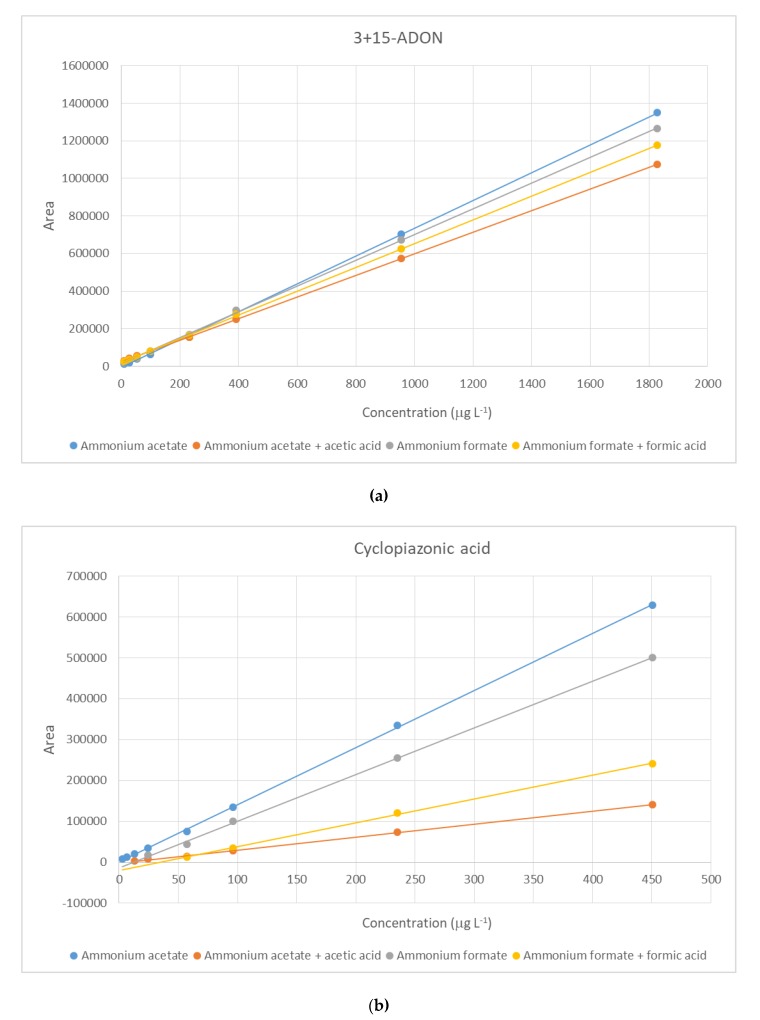

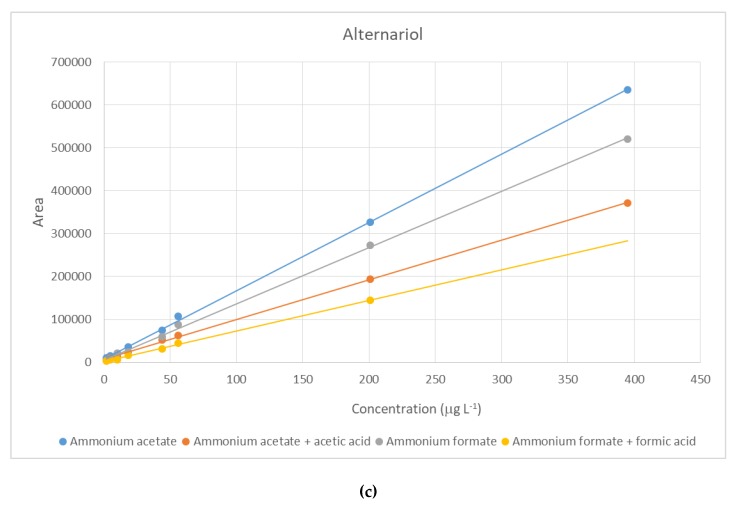
Response comparisons between the four mobile phase compositions: ammonium acetate 3mM, ammonium formate 3 mM and the addition of acetic or formic acid 0.1%, respectively for (**a**) 3+15 acetyldeoxynivalenol; (**b**) cyclopiazonic acid; and (**c**) alternariol.

Different mobile phase configurations were evaluated. Being the solvents used always water as aqueous phase and methanol as organic phase, two different buffers were tested (ammonium formate and ammonium acetate, 3 mM), as well as the addition of an acidic component (formic acid and acetic acid, 0.1% v/v). The regression equations for matrix-matched calibration using each mobile phase configurations are specified in Tables S1 and S2. As shown, although the slopes obtained using ammonium acetate and ammonium formate did not present great differences, some frequently found compounds gave better responses using ammonium acetate rather than ammonium formate. Such is the case of deoxynivalenol acetylated derivatives (3+15-ADON), FB_2,_ cyclopiazonic acid (CPA) or alternariol (AOH) (examples in Figure 1). Besides, the addition of an acidic component (formic or acetic acid) caused a substantial signal suppression in almost all cases in comparison with the mobile phase without acid. Exceptionally, few compounds such as andrastin A (AND A), and toxins T-2 and HT-2 gave better responses with the addition of 0.1% of acid. Therefore, the mobile phase configuration selected was a combination of water (A) and methanol (B), both containing 3 mM ammonium acetate. The method performance will be then assessed with this mobile phase composition.

The proposed chromatographic method offers a complete separation of all 26 mycotoxins and a good peak shape for quantification.

The only two exceptions were the isomers 3-DON and 15-DON for which the sum of the two metabolites was reported, as commonly done in previous published studies [29].

### 2.2. Method Performance Evaluation

**Figure 2:**
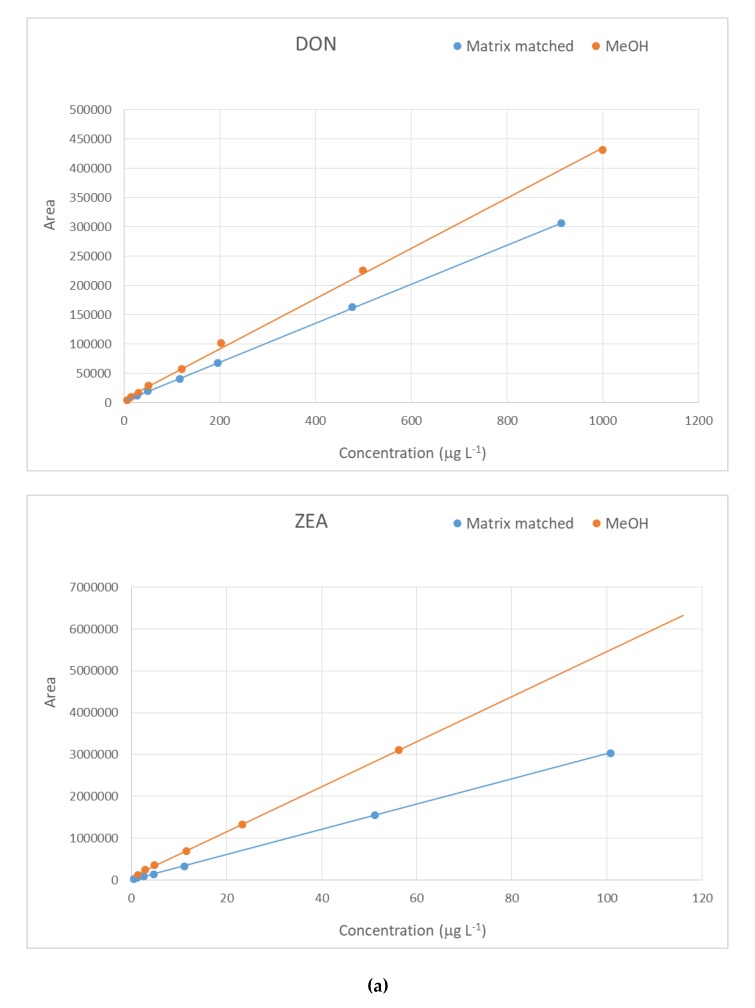

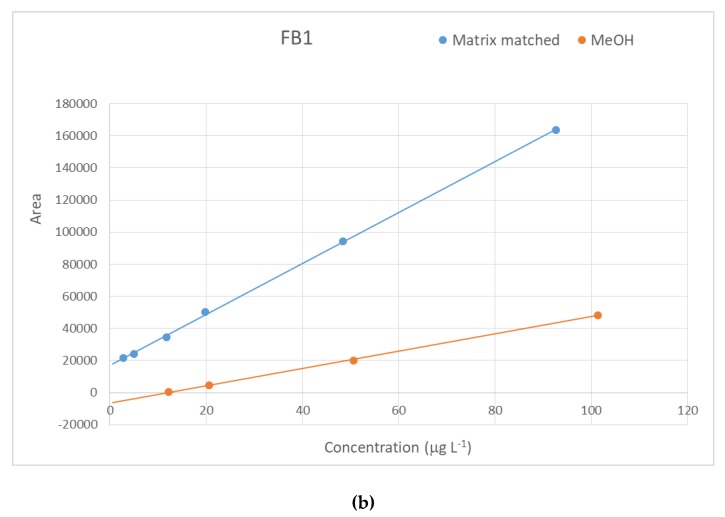
Matrix-matched calibration curves and external calibration. (**a**) Signal suppression: DON, ZEA; (**b**) signal enhancement: FB_1._

Mixed ration is a difficult matrix to work with because of the complexity of its composition. Thus, to achieve a reliable detection and quantification of mycotoxins, and exhaustive analytical methodology is needed, beginning with a matrix effect evaluation. Most publications working with this kind of matrix emphasized the need of a matrix-matched calibration in order to properly evaluate the concentration of the analytes [1,21]. For this purpose, a comparison was made between a matrix-matched standard calibration and an external calibration prepared in MeOH. The concentration range values covered depended on the mycotoxin, but in all cases, the range was of two orders of magnitude (see individual ranges of concentration in Table 1) with eight concentration levels. Matrix-matched calibration was performed using the sample with the lowest analyte content found (blank sample), spiked in the same range of concentrations as for the external calibration. For most compounds, the signal showed a suppressive effect when working with matrix (Figure 2), showing a median value of −35%, with some compounds (e.g., HT-2 toxin and AOH) exhibiting over 70% of signal suppression. However, there were a few examples of signal enhancement, such as the case of FB_1_ and FB_2_. Because of this variance, further studies and quantification were carried out using the matrix-matched approach.

Table 1 shows the determination coefficient (R^2^) for all analytes using matrix-matched calibration. R^2^ values were in all cases higher than 0.9991, showing an almost perfect linearity in the relation between the signals obtained and the concentration in the corresponding ranges. Both intra-day (n = 3) and inter-day (n = 3) precisions were calculated and expressed in terms of relative standard deviation (RSD) obtaining satisfactory values, ranging from 0.2% to 10.4%. The limits of quantification (LOQs) ranged from 0.3 to 50 ng mL^−1^.

To evaluate the accuracy of the methodology proposed, a multitoxin reference material containing ZEA, DON and fumonisins was extracted. Mixed ration is a complex and heterogeneous matrix and there is no related reference material available; therefore, a maize based one was used. The extraction recoveries achieved were 78.6% in the case of DON and 98.6% for ZEA, endorsing the trueness of the proposed methodology.

To evaluate the whole sample preparation and chromatographic methodology for the determination of all 26 mycotoxins, recovery studies were carried out using the same matrix as in the matrix-matched calibration. The results, expressed in percentages, are shown in Table 1, as well as the relative standard deviation in each case. Most compounds gave recovery values between 70% and 120%, especially the most frequently found in this kind of matrix, DON, ZEA, and FB_1_ and FB_2_. Only two mycotoxins achieved recoveries below 60%, at the two spiking levels (Enniatin B1 (ENN B_1_) and Penitrem A (PEN A)), but these analytes are not as recurrent and their contents in food/feed are not yet either regulated. The relative standard deviations were satisfactory since they were always lower than 9%.

ISO 21748:2017 provides recommendations [30] to assess the expanded measurement uncertainty (*U*).

Thanks to the performance parameters given in Table 1, especially the intra-day and inter-day precisions, the relative expanded measurement uncertainty (% *U*) could be calculated. As shown in Table 2 for those 17 mycotoxins that were detected in the mixed ration samples, % *U* were lower than 15% in all cases, except for α-ZOL.

### 2.3. Application to Real Samples

Using the proposed methodology, 97 mixed ration samples collected in one year at 20 different dairy farms from Galicia (NW Spain) were analyzed. The criteria used for the correct identification of the analytes, in addition to the retention time, were the parameters given by the high-resolution mass spectrometry such as the exact mass accuracy, accepting only a 5 ppm error. The isotopic profile of the peak found as well as the formula proposed by the software for the mass obtained provided valuable additional information for the identification. Finally, contrasting the high resolution MS/MS spectra experimentally obtained with the available library spectra enabled a definitive confirmation.

**Figure 3:**
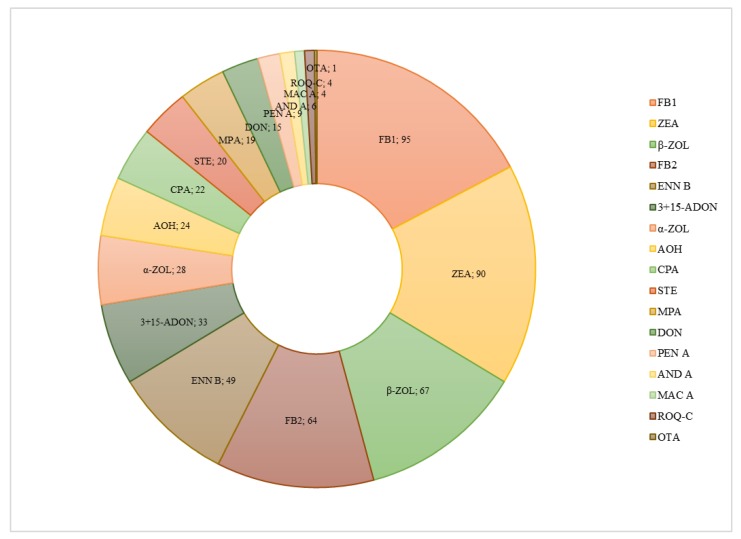
Total number of positives for each mycotoxin in the 97 samples analyzed.

Figure 3 summarizes the total of positives of each compound found in all samples. Fumonisins B_1_ and B_2_, zearalenone and its metabolite β-zearalenol, cyclopiazonic acid and enniatin B were the mycotoxins most frequently found. FB_1_ was detected in 95 samples; ZEA in 90 samples; ENN B in 49 samples and DON in 15 samples. This results are in consonance with other studies published on mycotoxin occurrence in feed [8,20,31]. All samples exhibited co-occurrence of two or more mycotoxins, repeatedly zearalenone and fumonisin B_1_, ZEA-FB_1_-ENN B and ZEA-FB_1_-DON. These co-occurrences are often found in feedstuff, as recent studies show [29,32]. However, the concentrations found in the mixed ration samples of the present study are much lower than those reported in these previous studies on maize based feed and silages [26,29,32].

Table 2 collects the concentration ranges and the average values in which the analyzed mycotoxins were present in the samples. ZEA, DON, FB_1_, and FB_2_ concentrations were always lower than the recommended values in feed intended for dairy cows; 0.5, 5, and 50 mg kg^−1^, respectively. It should be noted that 3+15-ADON concentrations were one magnitude order higher than DON ones, with an occurrence frequency of 34%. As stated by EFSA in 2013 [33], it is important to collect more data on the incidence of these derivatives to better characterize their potential contribution to the total exposure to DON.

ENN B showed an average concentration of 0.32 mg kg^−1^ and reached a maximum content of 1.3 mg kg^−1^. A particular attention should be paid to this cyclic depsipeptide since a recent EFSA scientific report, combining an in vivo toxicity and genotoxicity approach, concludes that ENN B poses a genotoxic hazard [34].

Finally, the screening of non-targeted mycotoxins conducted by implementing a retrospective approach with the same acquired data files and by contrasting HR MS/MS spectra with those of the mycotoxin library enabled to identify monocerin, aurofusarin, beauvericin, chlamydosporol, sambucinol, mevastatin, and two forms of enniantin, enniantins B4 and J1. Two anthraquinone derivatives, emodin and chrysophanol, were also identified.

## 3. Conclusions

The proposed analytical methodology based on QuEChERS with (d)SPE clean-up and HPLC-HRMS analysis allowed the screening and quantitation of 26 mycotoxins in mixed rations. The full method was successfully optimized, and its accuracy was confirmed using a reference material, as well. Recovery values were acceptable for most analytes, giving good results considering the complexity of the matrix for a multi-analyte methodology. Matrix-matched calibration was utilized to compensate for matrix effects, mostly suppressive, except from fumonisins.

The analytical method has been applied to the analysis of 97 samples. Fumonisins B_1_ (97%) and B_2_ (65%), zearalenone (92%), its metabolite β-zearalenol (68%) and enniatin B (50%) were the most frequently found mycotoxins. All samples presented co-occurrence of two or more mycotoxins, recurrently zearalenone and fumonisin B_1_, amongst others. The use of SWATH^TM^ acquisition mode proved to be a very attractive approach for the unambiguous identification as well as for the accurate quantification of the target mycotoxins in complex feed samples. Additional screening based on a retrospective approach enabled the identification of non-targeted mycotoxins, most of them originated from *Fusarium* fungi.

## 4. Materials and Methods

### 4.1. Reagents and Standards

The studied compounds, their molecular formula, exact mass, CAS numbers and structure are summarized in Table 3. Standards of 3+15-ADON, DON, HT-2, OTA, ROQ-C, T-2, ZEA, AFB_1_, AFG_1_, AFB_2_, AFG_2_ were obtained from Biopure (Romer Labs, Tulln, Austria); ENN B, ENN B_1_, STER, α-ZOL and β-ZOL from Sigma-Aldrich (Saint Louis, MO, USA); fumonisins FB_1_ and FB_2_ from Acros Organics (New Jersey, USA); MARC A and AND A from Santa Cruz Biotechnology (Dallas, TX, USA); CPA and MPA from Alpha Aesar (Ward Hill, MA, USA); AOH and PEN A from Cayman Chemical (Ann Harbor, MI, USA). Acetonitrile, water and methanol (both > 99.95% purity) were provided by Carlo Erba (Milan, Italy). Formic acid (99%), acetic acid glacial, ammonium formate and ammonium acetate were all LC-MS grade and were provided by Biosolve (Dieuze, France and Valkenswaard, The Netherlands).

A multitoxin maize reference material (QCM7C1, Biopure, Romer Labs, Tulln, Austria) was used for checking the extraction method accuracy.

Individual stock solutions for each mycotoxin were prepared in acetonitrile. Further dilutions and mixtures were prepared in methanol. All solutions were stored at −20 °C.

### 4.2. Sampling and Sample Treatment

Mixed rations for dairy cows in Galicia usually rely on own forages and concentrates, and in recent years, the ensiled maize proportion is being increasing. Concentrates are mostly a complement of protein and energy to balance dairy cow rations. In Galicia, these concentrates are made with soya cake (30%) and rapeseed cake (20%) to increase protein, and with cereals, mainly maize (25%) and barley (15%), to achieve the cattle energy requirement.

97 mixed ration samples collected in 20 dairy farms between October 2017 and October 2018 in Galicia (NW Spain). Five hundred grams of the sample was dried for 48 h at 40 °C and then put in vacuum bags and stored at 4 °C until their analysis.

### 4.3. QuEChERS Extraction

Two grams of unifeed samples were weighed and placed in 50 mL falcon tubes with the addition of 10 mL of water and 10 mL of acetonitrile/formic acid (90:10% v/v). The tubes were placed in an automated shaker for 1 h at room temperature. Then, a mixture of 0.5 NaSesquihydrate/1g NaCitrate/1g NaCl/4g MgSO_4_ (DisQuE^TM^ Pouch for CEN, Waters, Milford, USA) was added and the tube was shaken vigorously by hand for 1 min. After centrifugation (3398× *g* for 5 min), a portion of the supernatant was saved for subsequent clean-up.

### 4.4. Clean-Up Procedure

An Oasis PRiME HLB cartridge (3cc, 150 mg), Waters, Milford, MA, USA) was placed on a vacuum manifold. Without previous conditioning, after discarding 0.4 mL of supernatant, a 1 mL aliquot was passed-trough the cartridge and, the collected extract was then transferred to a 2 mL dSPE tube containing a mixture of sorbents (150 mg MgSO_4_, 50 mg PSA, 30 mg C18, 30 mg Al-N) (Waters, Milford, USA). After centrifugation (2 min at 2360× *g*), a 500 µL aliquot was taken, evaporated under a gentle nitrogen stream, and reconstituted in 350 µL of methanol.

### 4.5. HPLC-QTOF Analysis

For LC-HRMS analysis, a HPLC system (Shimadzu Nexera X2) consisting of two high-pressure pumps (LC-30AD) and a SIL-30AC autosampler was used. HRMS detection was performed by a SCIEX (Ontario, Canada) TripleTOF^®^ 5600+ equipped with a DuoSprayTM ion source and an Electrospray Ionization (ESI) probe. The chromatographic separation was achieved with a Kinetex bioZenTM Peptide XB-C18 (50 × 2.1 mm, 2.6 μm) column from Phenomenex (Torrance, CA, USA), kept at 40 °C with a CTO-30A column oven.

The mobile phase consisted of water (A) and methanol (B), both buffered with 3mM ammonium formate or ammonium acetate. Formic acid and acetic acid addition (0.1%) was also tested in combination with its respective buffer.

An 8-min gradient elution profile (10% B to 100% B) was employed reaching a total runtime of 15 min. The mobile phase flow-rate was kept to 0.25 mL mL^−1^ and the injection volume was set at 10 µL.

Regarding the HRMS conditions, the source temperature was set at 550 °C, the ion source gas at 50 (au, arbitrary units), the curtain gas at 30 (au), and the ion spray voltage floating at 5500 V (−4500 V in the negative mode).

The HRMS workflow consisted of a Full Scan, using 250 ms as accumulation time and 80 V (−80 V in negative mode) as the declustering potential in the ESI. Simultaneously, a data independent approach based on SWATH (Sequential Windowed Acquisition of All Theoretical Fragment Ion Mass Spectra) was performed. A wide mass range (80–850 Da) was divided in 30 mass windows with an accumulation time of 35 ms for each one. The cycle time was slightly higher than 1 s, enabling a perfect reconstruction of any chromatographic peak. The declustering potential was set to 80 V (−80 V in negative mode) and the collision energy was 40 V (−40 V in negative mode), with an energy spread of 20 V.

SWATH acquisition is a Data Independent Acquisition (DIA) strategy that delivers the complete picture of a sample. It provides the best method for performing sample analysis, identifying and quantifying every detectable analyte in a single chromatographic run.

In contrast to traditional mass spectrometry acquisition techniques that rely on Data Dependent Acquisition (DDA) strategies, SWATH acquisition is not “dependent” upon some pre-set criteria determined by the abundance of the compound. With SWATH acquisition, every detectable ion, irrespective of its concentration, is fragmented, identified and quantified to provide the high-resolution full MS and MS/MS picture for every peak. The data produced by SWATH then contains all the information that would be acquired in a multitude of different experimental approaches in a single all-encompassing dataset. Furthermore, SWATH acquisition cycle time does not increase as the number of target compounds increases.

In terms of post-acquisition, the complete set of data can serve as a digital archive of each sample, allowing full access to pull out anything that you may be interested in, without the need to re-inject your samples. Should new hypotheses arise in the future, this feature allows you to re-interrogate the sample data without re-analyzing the actual sample.

The system was operated by Analyst^®^ 1.7.1 (SCIEX, Ontario, Canada) control software, PeakView^®^ 2.2 (SCIEX, Ontario, Canada) processing software and MultiQuant^®^ 3.0 (SCIEX, Ontario, Canada) quantitation software. A mycotoxin HR MS/MS spectral library (1.0), containing 288 compounds was used for identification and confirmation purposes.

As usual, mass accuracy is expressed in ppm, calculated as the relative difference between the measured mass and the actual mass of each analyte, as follows:

$$Accuracy\;\left(ppm\right)=\frac{\left(\mathrm{Measured}\;\mathrm{mass}-\mathrm{Actual}\;\mathrm{mass}\right)}{\mathrm{Actual}\;\mathrm{mass}}*1000000$$

### 4.6. Method Validation

The limit of quantification (LOQ) of each analyte was evaluated using the parameters of each matrix-matched calibration curve and the S/N obtained for the lowest concentration level of each analyte.

The matrix effect was calculated by subtracting the ratio of the slope values of the matrix-matched calibration curve and the external calibration curve to 1, and expressing it as a relative percentage (%) (See Table 1).

The mixed ration sample selected for recovery experiments was spiked in triplicate at two concentration levels (individual values indicated in Table 1). Aliquots of 2 g of fortified mixed ration samples were let interacting for 20 h at ambient temperature and protected from natural and artificial light exposure. Recovery values were given as the ratio of the signal obtained for these fortified samples and the response of the corresponding concentration level in the matrix-matched standards.

ISO 21748:2017 provides recommendations [30] to assess the expanded measurement uncertainty (*U*) which was calculated (see following equations) by multiplying the combined standard uncertainty (*u*_c_) by a coverage factor (*k*). At a confidence level of 95%, this *k* factor was set to two. The standard uncertainty (*u*_c_) formulae combined the variance of the inter-day precision (S^2^_R_) and the uncertainty associated with the bias (*u_bias_*^2^). The latter was calculated using the number of replicates (*n*), the number of different conditions (*p*), the variance of the intra-day precision (S^2^r) and the variance of the inter-day precision (S^2^_R_).

$$ubias=\sqrt{\left(\frac{{S^{2}}_{R}-\left(1-\frac{1}{n}\right){S^{2}}_{r}}{p}\right)}\;uc=\sqrt{{S^{2}}_{R}+ubias2}\;U=ky\;uc$$

## Figures and Tables

**Table 1:** HPLC-Triple time-of-flight (TOF) performance for the mycotoxin analysis: linearity, intra and inter-day precision, detection limits, recovery and matrix effect.

Compounds	R^2^	Linear Range (ng mL^−1^)	Intra-Day Precision (%) (n = 3)	Inter-Day Precision (%) (n = 3)	LOQs (ng mL^−1^)	Recoveries				Matrix Effect (%)
						Concentration (ng mL^−1^)	Recovery (%) (% RSD)	Concentration (ng mL^−1^)	Recovery (%) (% RSD)	
**3+15-ADON**	0.9998	10.7–1828.3	2.4	5.8	24	339.6	85.8 (3.9)	95.4	90.5 (3.0)	−0.6
**DON**	0.9999	26.7–912.8	2.4	6.2	41.1	169.6	114.2 (0.8)	47.7	121.9 (8.7)	−22.4
**ENN B**	0.9999	40.6–905.4	2.5	5.9	46.8	168.2	88.8 (4.1)	47.6	132.6 (5.8)	33.9
**ENN B_1_**	1.0000	13.5–479.7	1.1	1.4	24	170.6	58.3 (1.2)	47.9	67.4 (3.5)	−29.6
**FB_1_**	0.9996	2.7–92.6	1.7	5.2	2.9	17.2	92.7 (2.2)	4.8	29.6 (6.6)	195.3
**FB_2_**	0.9998	1.3–85.8	0.2	0.4	2.4	15.9	118.1 (0.3)	4.5	98.7 (4.2)	197.8
**HT-2**	0.9998	1.1–190.7	2.3	2.2	3.3	35.4	98.9 (0.4)	10.0	61.3 (2.0)	−69.8
**MAC A**	0.9998	2.6–87.9	3.1	6.0	4.2	16.3	104.0 (5.5)	4.6	70.4 (2.9)	−51.2
**OTA**	0.9991	1.6–36.4	4.8	5.2	2.0	6.8	71.4 (5.2)	1.9	54.8 (1.1)	−49.9
**ROQ-C**	0.9998	0.2–36.1	2.5	2.9	0.6	6.7	32.2 (2.5)	1.9	87.0 (8.0)	−31.0
**STE**	0.9996	0.5–35.9	4.7	5.1	1.2	6.7	80.9 (3.0)	1.9	76.3 (9.1)	−56.3
**T-2**	0.9999	0.4–9.1	0.9	1.1	0.5	1.7	155.2 (1.9)	0.6	85.7 (0.3)	−28.8
**CPA**	0.9998	2.6–450.5	1.1	3.8	7.0	83.7	53.7 (0.3)	23.5	83.4 (5.5)	−25.9
**AND A^1^**	0.9999	0.1–19.1	1.2	1.3	0.3	3.5	78.2 (2.5)	0.9	97.6 (3.0)	−53.0
**AOH** **^1^**	0.9996	2.0–394.7	3.7	5.6	3.5	73.1	66.7 (2.1)	18.6	84.2 (3.0)	−89.6
**MPA^1^**	0.9999	1.4–33.2	4.1	3.6	1.6	6.1	81.4 (1.8)	1.7	101.0 (1.1)	−34.6
**PEN A**	0.9993	1.0–191.3	1.4	4.0	2.1	35.4	37.8 (0.5)	9.0	56.5 (0.2)	−11.4
**α-ZOL^1^**	0.9999	2.5–249.2	4.9	9.4	5.7	90.8	95.8 (6.5)	23.2	90.5 (1.7)	−3.0
**β-ZOL^1^**	0.9999	2.6–256.4.4	4.8	5.2	5.7	93.4	77.9 (5.2)	23.8	108.2 (4.2)	−50.0
**ZEA^1^**	0.9999	0.5–100.7	4.6	4.8	1.2	18.7	83.4 (5.9)	4.8	103.7 (5.9)	−44.0
**FUS X**	0.9995	2.0–395.3	0.6	0.6	6	73.2	96.4 (0.7)	18.7	109.6 (0.9)	25.0
**AF B_1_**	0.9998	0.1–103.5	4.4	10.4	0.3	21.3	103.3 (5.2)	11.4	57.8 (8.9)	−65.0
**AF B_2_**	1.0000	0.4–25.9	4.7	4.4	1.2	5.3	105.2 (2.9)	2.9	62.5 (7.6)	−43.2
**AF G_1_**	0.9998	0.1–103.5	6.8	10.1	0.3	21.3	96.5 (1.9)	11.4	49.2 (6.2)	−36.3
**AF G_2_**	0.9998	0.4–25.9	4.6	4.3	1.2	5.3	118.3 (0.6)	2.9	135.5 (0.4)	−30.3

^1.^ negative mode.

**Table 2:** Concentration ranges, average, maximum and minimum concentrations (with relative expanded uncertainty, *U* (%)) and occurrence frequency of the 17 mycotoxins found in the 97 mixed ration samples.

Compounds	Concentration (ng g^−1^)				N° Positive	Occurrence Frequency (%)
	*Average*	*Max*	*Min*	*U* (%)		
**3+15-ADON**	426.9	770.0	214.1	*13.2*	33	33.7
**DON**	52.8	81.3	36.5	*14.1*	15	16.3
**ENN B**	322.0	1305.5	53.1	*13.4*	49	50.0
**FB_1_**	578.8	1453.9	14.0	*11.6*	95	96.9
**FB_2_**	160.6	481.1	13.3	*1.1*	64	65.3
**MAC A**	124.3	209.9	46.1	*13.6*	4	4.1
**OTA**	5.1	5.1	5.1	*11.0*	1	1.0
**ROQ-C**	36.9	59.3	19.6	*6.3*	4	4.1
**STER**	8.7	15.3	2.6	*10.9*	20	20.4
**CPA**	96.5	257.0	2.6	*8.7*	22	22.4
**AND A**	33.3	91.0	8.8	*2.8*	6	6.1
**AOH**	126.3	295.4	12.7	*12.4*	24	24.5
**MPA**	262.2	3151.4	8.2	*7.4*	19	19.4
**PEN A**	159.7	234.5	63.7	*9.1*	9	9.2
**α-ZOL**	32.5	50.3	25.3	*21.1*	28	28.6
**β-ZOL**	35.3	78.9	8.1	*11.0*	67	68.4
**ZEA**	10.2	23.2	3.5	*10.2*	90	91.8

**Table 3:** Target mycotoxins: molecular formula, exact mass, CAS number, molecular ion, retention time and structure.

Mycotoxins	Acronym	Molecular Formula	Exact Mass (m/z)	CAS Number	Molecular Ion	RT (min)	Structure
**3-Acetyl deoxynivalenol**	3-ADON	C_17_H_22_O_7_	338.1365	50722-38-8	[M+H]^+^	3.83	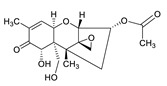
**15-Acetyl deoxynivalenol**	15-ADON	C_17_H_22_O_7_	338.1365	88337-96-6	[M+H]^+^	3.83	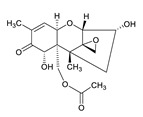
**Deoxynivalenol**	DON	C_15_H_20_O_6_	296.1259	51481-10-8	[M+H]^+^	2.24	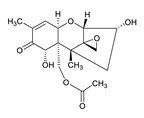
**Enniatin B**	ENN B	C_33_H_57_N_3_O_9_	639.4094	917-13-5	[M+H]^+^	8.15	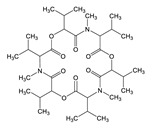
**Enniatin B_1_**	ENN B_1_	C_34_H_59_N_3_O_9_	653.4251	19914-20-6	[M+H]^+^	8.30	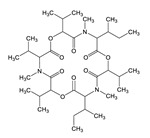
**Fumonisin B_1_**	FB_1_	C_34_H_59_NO_15_	721.3884	116355-83-0	[M+H]^+^	5.20	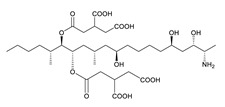
**Fumonisin B_2_**	FB_2_	C_34_H_59_NO_14_	705.3935	116355-84-1	[M+H]^+^	6.20	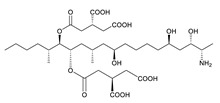
**HT-2 toxin**	HT-2	C_22_H_32_O_8_	424.2097	26934-87-2	[M+NH_4_]^+^	5.70	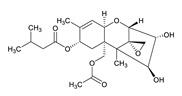
**Marcfortine A**	MAC A	C_28_H_35_N_3_O_4_	477.2627	75731-43-0	[M+H]^+^	6.97	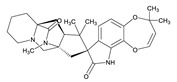
**Ochratoxin A**	OTA	C_20_H_18_ClNO_6_	403.0822	303-47-9	[M+H]^+^	5.36	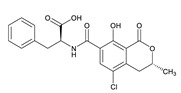
**Roquefortine C**	ROQ-C	C_22_H_23_N_5_O_2_	389.1851	58735-64-1	[M+H]^+^	6.50	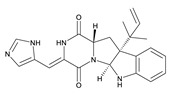
**Sterigmatocystin**	STE	C_18_H_12_O_6_	324.0633	10048-13-2	[M+H]^+^	6.62	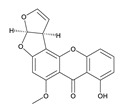
**T-2 toxin**	T-2	C_24_H_34_O_9_	466.2202	21259-20-1	[M+NH_4_]^+^	6.16	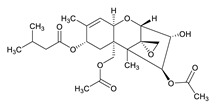
**Cyclopiazonic acid**	CPA	C_20_H_20_N_2_O_3_	336.1473	18172-33-3	[M+H]^+^	5.00	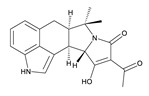
**Andrastin A**	AND A	C_28_H_38_O_7_	486.2617	174232-42-9	[M-H]^-^	5.95	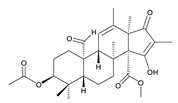
**Alternariol**	AOH	C_14_H_10_O_5_	258.0528	641-38-3	[M-H]^-^	5.64	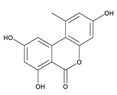
**Mycophenolic acid**	MPA	C_17_H_20_O_6_	320.1259	24280-93-1	[M-H]^-^	4.93	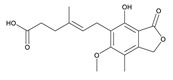
**Penitrem A**	PEN A	C_37_H_44_ClNO_6_	633.2857	12627-35-9	[M+H]^+^	7.64	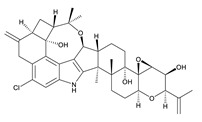
**α-Zearalenol**	α-ZOL	C_18_H_24_O_5_	320.1623	36455-72-8	[M+H]^+^	5.95	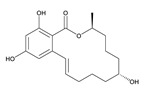
**β-Zearalenol**	β-ZOL	C_18_H_24_O_5_	320.1623	71030-11-0	[M+H]^+^	6.34	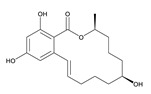
**Zearalenone**	ZEA	C_18_H_22_O_5_	318.1467	17924-92-4	[M+H]^+^	6.48	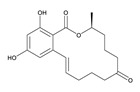
**Fusarenon X**	FUS X	C_17_H_22_O_8_	354.1314	23255-69-8	[M+H]^+^	3.03	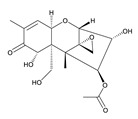
**Aflatoxin B_1_**	AF B_1_	C_17_H_12_O_6_	312.0633	1162-65-8	[M+H]^+^	4.95	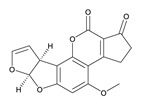
**Aflatoxin B_2_**	AF B_2_	C_17_H_14_O_6_	314.0790	7220-81-7	[M+H]^+^	4.75	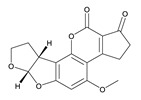
**Aflatoxin G_1_**	AF G_1_	C_17_H_12_O_7_	328.0583	1165-39-5	[M+H]^+^	4.54	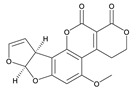
**Aflatoxin G_2_**	AF G_2_	C_17_H_14_O_7_	330.0739	7241-98-7	[M+H]^+^	4.33	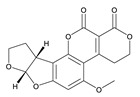

## Supplementary Materials

The following are available online at https://www.mdpi.com/2072-6651/12/3/206/s1, Table S1: Regression equations for matrix-matched calibrations of the target mycotoxins in positive mode, using different mobile phase compositions. Table S2: Regression equations for matrix-matched calibrations of some target mycotoxins in negative mode, using different mobile phase compositions.
