# Supplementary material for: Combined (d)SPE-QuEChERS Extraction of Mycotoxins in Mixed Feed Rations and Analysis by High Performance Liquid Chromatography-High-Resolution Mass Spectrometry
Source: Toxins (Basel). 2020 Mar 23;12(3):206. doi: 10.3390/toxins12030206 (PMC7150789; doi:10.3390/toxins12030206)
Supplement: Supplementary file 1 [file toxins-12-00206-s001.pdf]

Supplementary Materials: Combined (d)SPE-QuEChERS Extraction of Mycotoxins in Mixed Feed Rations and Analysis by High Performance Liquid Chromatography-High-Resolution Mass Spectrometry

Rocio Facorro, Maria Llompart and Thierry Dagnac

Table S1. Regression equations for matrix-matched calibrations of the target mycotoxins in positive mode, using different mobile phase compositions.

Compounds	Ammonium formate 3mM	Ammonium formate 3mM + formic acid 0.1%	Ammonium acetate 3mM	Ammonium acetate 3mM + acetic acid 0.1%
3+15-ADON	y = 686.6x + 13570	y = 632.5x + 20095	y = 741.3x - 6619.8	y = 575.3x + 23525
DON	y = 430.2x - 4372.4	y = 364.6x - 5441.5	y = 332.9x + 2719.6	y = 434.5x - 13436
ENN B	y = 2462.5x + 18338	y = 783.0x + 27600	y = 1897.4x + 14917	y = 795.5x + 7306.4
ENN B_1	y = 8491x + 27371	y = 3469.3x + 17885	y = 6296.3x + 4815.1	y = 3413.2x + 24601
FB_1	y = 1819.1x + 15571	y = 1629x + 15294	y = 1585.2x + 17131	y = 1241.4x + 18913
FB_2	y = 1748.4x + 9814	y = 1287.5x + 9663.2	y = 1770.2x + 3537.9	y = 1292.5x + 8063.2
HT-2	y = 654.3x - 5538	y = 616.1x + 11592	y = 546.6x + 11334	y = 651.5x + 12973
MAC A	y = 5046.2x + 27423	y = 3313.5x + 15555	y = 4233x + 34351	y = 3370.6x + 10198
OTA	y = 949.4x + 2287.3	y = 1080.5x - 313.4	y = 914.4x - 716.28	y = 788.2x - 2115.5
ROQ-C	y = 2481.1x - 1376.4	y = 267.7x + 1894.4	y = 2063.3x + 3143.5	y = 825.9x - 692.23
STE	y = 2474.5x - 49.734	y = 2327.9x - 990.45	y = 2368.8x - 640.3	y = 1988x + 1019.4
T-2	y = 4047.8x + 7194.4	y = 4099.9x + 9487.3	y = 4514.8x + 2602	y = 3734.1x - 279.64
CPA	y = 1143x - 13643	y = 581.1x - 19926	y = 1395.3x + 1617.6	y = 316.7x - 1866.3
AND A	y = 1809.1x + 8567.3	y = 970.2x + 9503.1	y = 1397x + 6185.6	y = 2383.2x – 2540.3
AOH	y = 132.2x – 1451.3	y = 79.7x + 1439.5	y = 124.4x – 1007.5	y = 71.3x – 182.9
MPA	y = 2150.4x + 945.9	y = 4329.2 + 10702	y = 2168.3x – 399.3	y = 3989.1x + 9532.2

**PEN A**	y = 1096.6x - 672.95	y = 671.4x - 1002.7	y = 1137.6x + 1878.5	y = 702.5x + 1802.5
**α-ZOL**	y = 4635.3x + 65293	y = 6545.7x + 174983	y = 9769x + 21991	y = 4653.6x + 148058
**β-ZOL**	y = 3823x + 52497	y = 2121.8x + 63093	y = 3326.5x + 20531	y = 2151.9x + 63908
**ZEA**	y = 28533x + 23121	y = 24249x + 13997	y = 30168x + 1236.6	y = 23553x + 25766
**FUS X**	y = 318.2x + 17780	y = 325.3x + 6505.9	y = 476.0x + 2772.7	y = 245.8x + 3309.3
**AF B_1**	y = 7189.9x + 3491.5	y = 5841.8x + 109544	y = 4188.4x + 98650	y = 5364.7x + 983.2
**AF B_2**	y = 7472.3x - 5431	y = 4863.8x + 31476	y = 6580.1x + 3935.8	y = 4994.4x - 7080.3
**AF G_1**	y = 5555.5x + 3427.9	y = 5117.5x + 5375.1	y = 4811.3x + 1885	y = 3090x + 5023.2
**AF G_2**	y = 5981x + 42106	y = 4215.6x + 33050	y = 5137.3x + 15877	y = 2822.9x + 23334

Table S2. Regression equations for matrix-matched calibrations of some target mycotoxins in negative mode, using different mobile phase compositions.

Compounds*	Ammonium formate 3mM	Ammonium formate 3mM + formic acid 0.1%	Ammonium acetate 3mM	Ammonium acetate 3mM + acetic acid 0.1%
**3+15-ADON**	y = 30.26x + 8404.1	y = 13.84x + 349.7	y = 331,9x + 22108	y = 132.3x + 6379.2
**DON**			y = 106.4x - 2629.5	y = 67.92x + 701.6
**FB_1**	y = 2727.1x + 28409	y = 1318.5x + 12483	y = 2322.4x + 17661	y = 1525.9x + 9609.3
**FB_2**	y = 2635.8x + 12000	y = 1490.1x + 6757	y = 2777.2x + 8039.4	y = 1290.7x + 10677
**MAC A**			y = 615.2x + 2850.2	y = 381.7x + 36369
**OTA**	y = 2500.6x - 542.8	y = 1989.4x - 479.03	y = 3107.4x - 9301	y = 2487.8x + 419.52
**ROQ-C**	y = 969.9x + 5386.6	y = 793.4x + 2059.9	y = 1276.1x + 13461	y = 585.6x + 5043.5
**STE**		y = 315.3x + 3112.7		
**CPA**	y = 1353x - 86.73	y = 817.2x + 8916.4	y = 1943.1x + 73304	y = 1075.7x + 32130
**AND A**	y = 23861x + 35680	y = 27889x + 10102	y = 20736x + 6471,2	y = 17590x + 17678
**AOH**	y = 1311.8x + 5029.6	y = 714.11x + 2167.1	y = 1590.4x + 8158.5	y = 924.8x + 7135.2
**MPA**	y = 2322.4x + 1374.3	y = 2861.6x + 1540.6	y = 3856x + 146.64	y = 1718.3x + 22040
**PEN A**	y = 1446x + 1127	y = 606.9x + 1153.7	y = 1576.6x + 1038.4	y = 700.77x + 1424.5
**α-ZOL**	y = 44948x + 313726	y = 57428x + 169498	y = 64727x + 584143	y = 38802x + 352390
**β-ZOL**	y = 35230x + 282359	y = 17684x + 184993	y = 32507x + 230167	y = 25063x + 279511
**ZEA**	y = 111740x + 74995	y = 83703x + 49378	y = 127924x + 79337	y = 87939x + 81232

*ENN B, ENN B1, HT-2, T-2, FUS X and aflatoxins did not give exploitable calibration curves in negative mode.
